# An Assassin among Predators: The Relationship between Plant-Ants, Their Host Myrmecophytes and the Reduviidae *Zelus annulosus*

**DOI:** 10.1371/journal.pone.0013110

**Published:** 2010-10-01

**Authors:** Messika Revel, Alain Dejean, Régis Céréghino, Olivier Roux

**Affiliations:** 1 CNRS, UMR 8172, Ecofog (Écologie des Forêts de Guyane), Campus agronomique, BP 709, Kourou, France; 2 Université de Toulouse, UPS, INPT, EcoLab, Toulouse, France; 3 CNRS, UMR 5245, EcoLab (Laboratoire d'Ecologie Fonctionnelle), Toulouse, France; University of Plymouth, United Kingdom

## Abstract

Tropical plants frequently live in association with ants that protect their foliage from defoliators. Among them, myrmecophytes have evolved mutualisms with a limited number of plant-ants that they shelter and feed, and, in return, benefit from some protection. *Hirtella physophora* (Chrysobalanaceae), for example, houses *Allomerus decemarticulatus* (Myrmicinae) that build gallery-shaped traps to catch large prey. In French Guiana, we frequently observed the assassin bug *Zelus annulosus* (Reduviidae, Harpactorinae) on the leaves of *H. physophora*. Here, we studied the distribution of *Zelus annulosus* among understory plants in the Guianese rainforest and found it only on pubescent plants, including *H. Physophora*, whether or not it was sheltering an *A. decemarticulatus* colony, but only rarely on other myrmecophytes. The relationship between *Z. annulosus* and its host plants is, then, also mutualistic, as the plant trichomes act as an enemy-free space protecting the nymphs from large predatory ants, while the nymphs protect their host-plants from herbivorous insects. Through their relationship with *A. decemarticulatus* colonies, *Z. annulosus* individuals are protected from army ants, while furnishing nothing in return. In those cases where *H. physophora* sheltered both an *A. decemarticulatus* colony and *Z. annulosus* nymphs, certain plant individuals repeatedly sheltered nymphs, indicating that female bugs may select not only pubescent plants but also particular *H. physophora* treelets having characteristics more favourable to the development of their progeny.

## Introduction

Tropical plants frequently live in association with ants that protect their foliage from defoliators. Among them, myrmecophytes have evolved mutualisms with a limited number of plant-ants that they shelter in domatia (i.e., hollow branches or thorns and leaf pouches) and usually provide with food through extra-floral nectar and/or food bodies. In return, plant-ants protect their host myrmecophytes from defoliators, competitors and pathogens [1]. Also, many plant-ants attend sap-sucking hemipterans for their honeydew that do not proliferate since the ants consume a part of them [2], [3].

As the transmission of ant-myrmecophyte mutualisms is horizontal (i.e., new treelets need to be colonized by a founding queen ant), other insect species can short-circuit these associations before ant colonization, exploiting the rewards provided by the plant whilst providing nothing in return [1]. Most of them - including not only ants, but also heteropterans, coleopterans and lepidopterans [4]–[10] - are parasites of the mutualism or exploiters with no mutualistic ancestor. Although plant-ants are very aggressive toward alien arthropods likely to be found on their host myrmecophytes, some species of spiders, coleopterans and lepidopterans are tolerated [6], [11], and social and solitary wasps can even install their nests on myrmecophytes. They benefit from protection from army ants, but provide nothing in return to the myrmecophyte or to the plant-ant, resulting in a form of commensalism [12], [13]. Ant colonies can also tolerate commensalists such as isopods, millipedes, collembolans, orthopterans, lepidopterans and coleopterans [14].

In French Guiana, we frequently observed the assassin bug *Zelus annulosus* (Reduviidae, Harpactorinae) on the leaves of understory plants. Indeed, some Harpactorinae species live in a specific relationship with certain plant species from which they obtain carbohydrates from food bodies, extrafloral nectar and hemipteran honeydew, or from sap by biting the plants [4], [15]–[17]. Other kinds of specific interactions with plants are indirect; for instance, some reduviids prey on insects associated with a particular plant species [18]–[20]. In fact, most reduviid sub-families are composed of arthropod predators, including those specialising in the predation of ants, termites, or diplopods [21], [22]. Also, while most Harpactorinae species secrete a sticky substance that coats their legs, facilitating prey capture, others have to gather such substances from specific plants on which they are frequently found [18], [20], [23].

Among those plants on which we observed *Z. annulosus* was *Hirtella physophora* (Chrysobalanaceae), a myrmecophyte that specifically houses colonies of the plant-ant *Allomerus decemarticulatus* (Myrmicinae) in pouches situated at the base of the leaf lamina, and provides them with extrafloral nectar. The workers of this species forage for prey on the host plant foliage and build gallery-shaped traps to capture large insects [24], [25]. Therefore, in the case of *H. physophora*, two predators seem to share a limited territory represented by the plant's foliage.

The assumption that *Z. annulosus* is able to coexist with *A. decemarticulatus* raises questions about the nature of the biological interactions between *Z. annulosus*, understory plants and ants patrolling their foliage for prey [26]. In general, these plants benefit from the presence of ants that patrol their foliage, and prey on herbivorous insects. The relationship is mutualistic when the plants reward the ants with sugary substances and/or shelter for the colony (as is the case for myrmecophytes).

To determine what the relationship between *Z. annulosus* and plants is, we needed to know if the plants benefit from some protection from defoliators, and if *Z. annulosus* individuals feed on sugary rewards or on the sap, or benefit in some other way. In the relationship between ants and *Z. annulosus*, we might wonder if there is only competition for prey, if one preys on the other (or if they prey on each other), or if a combination of competition and predation occurs as in typical intraguild predation [27].

In this study, we used a combination of field observations and controlled experiments to understand the patterns of host plant selection by *Z. annulosus*, and, subsequently, the nature of the relationships between *Z. annulosus*, understory plants, and ants. Our specific questions were: (1) Does *Z. annulosus* feed on the sap or extrafloral nectar of the host plant, (2) is *Z. annulosus* frequently found on myrmecophytes, (3) in the latter case, is *Z. annulosus* preyed upon by plant-ants associated with the myrmecophytes, or, on the contrary, does it prey on them or compete with them, (4) is the presence of *Z. annulosus* on *H. physophora* foliage detrimental to the plant and/or to its guest ants?

## Materials and Methods

### Ethics Statement

This study was conducted according to relevant national and international guidelines.

### Study site

This study was conducted from April 2009 to May 2010 at ten different sites in French Guiana where we had already conducted research on the distribution of four myrmecophytes growing in the understory: *H. physophora* (Chrysobalanaceae), *Cordia nodosa* (Boraginaceae), *Tococa guianensis* and *Maieta guianensis* (both Melastomataceae) [28]. One site was situated near Kourou at *la Montagne des singes* (5°04′19′′ N; 52°41′42′′ W), six others around the field station at *Petit Saut*, Sinnamary (5°03′39′′ N; 53°02′36′′ W), and the two remaining sites were located between the two previous sites along Route No. 1 (kilometric points 74 and 81).

### *Zelus annulosus*-plant interactions

We inspected a total of 901 *H. physophora* and examined 10 treelets ( = control plants; not taller than 2 m) situated within a radius of 5 m around each *H. physophora*. While doing so, we also examined 112 *C. nodosa*, 135 *T. guianensis*, and 149 *M. guianensis*. For each plant examined, we recorded the presence (or absence) of *Z. annulosus* individuals at all developmental stages, exuvia and egg clusters; the two latter are always situated under the leaves. Egg clusters are circular and protected by sticky threads constituting a web that likely wards off ants and other predators.

Because we found up to four egg clusters on the same plant and because we noted that certain plant individuals very frequently sheltered *Z. annulosus* nymphs, we verified if oviposition by females was not associated with the selection of a plant. To do so, we noted which *H. physophora* treelets (all tagged) sheltered *Z. annulosus* nymphs and/or egg clusters during seven surveys separated by ca. 3 months at one site situated at Petit Saut.

### *Zelus annulosus* on *Hirtella physophora*

To determine if vegetative traits and/or association with characteristic ants could be related to the presence of *Z. annulosus* on *H. physophora* treelets, we recorded the size of each plant, the number of leaves, and the presence or absence of an associated *A. decemarticulatus* colony. Because some Harpactorinae complement their diet by exploiting extrafloral nectaries or biting the plant to suck the sap [15], [17], each time we found *Z. annulosus* on a *H. physophora* treelet, we observed their behaviour during 15 minutes and noted if they bit the leaves and the position of the extremities of their rosters on the extrafloral nectaries.

We also noted what kinds of interactions occurred between *Z. annulosus* and *A. decemarticulatus* workers. During observations conducted prior to this study, we verified if one of the species preyed on the other. We then verified if they competed for prey by conducting experiments where we placed live *Nasutitermes* sp. termite workers at three different places on a plant sheltering both an *A. decemarticulatus* colony and *Z. annulosus* second or third instar nymphs. (1) We dropped a termite from ca. 5 cm in height onto the centre of the upper surface of the leaves where a *Z. annulosus* was hunting. (2) We then dropped a termite near the domatia where workers were much more numerous. (3) Finally, using forceps and selecting an internode situated above a leaf on which a *Z. annulosus* was hunting, we placed the prey on the side of the stem without the trap built by *A. decemarticulatus* workers. Each time we noted whether a *Z. annulosus* or an *A. decemarticulatus* worker caught the prey first and verified if the other tried to rob the prey, or if they shared it. Fifty replicates were conducted in each case.

### Effectiveness of the predatory behaviour of *Zelus annulosus* nymphs

We tested small (second and third instars; length<1.5 cm) and large (fourth and fifth instars; length≥1.5 cm) *Z. annulosus* nymphs reared in plastic boxes in the laboratory. Prior to the experiments, the nymphs were starved during 24 h. Using smooth forceps, we gently deposited one nymph onto a leaf taken back to the laboratory just prior to the experiment. After 5 minutes, we furnished a termite worker (*Nasutitermes* sp.) as prey to both small and large nymphs (56 and 28 cases, respectively) and a ca. 2.5-cm-long grasshopper only to large nymphs (30 cases). After 15 minutes, we noted if the prey was captured and bitten.

### Statistical analysis

For statistical comparisons of the number of termites caught successfully by *Z. annulosus* and *A. decemarticulatus* and comparisons of the proportion of trees bearing *Z. annulosus*, we used Fisher's exact-test. We adjusted appropriate probabilities for the number of simultaneous tests using the sequential Bonferroni procedure when necessary [29]. A Generalized Linear Model (GLM), with the Poisson distribution option, was used to investigate which factors (presence/absence of ants, number of leaves and size of trees) might be responsible for the presence of *Z. annulosus* on *H. physophora* treelets. All variables and their interactions were taken into account. All statistics were conducted using R 2.8.1 (R Development Core Team, 2008).

## Results

### Distribution of *Zelus annulosus* on plants of the understory

**Figure 1:**
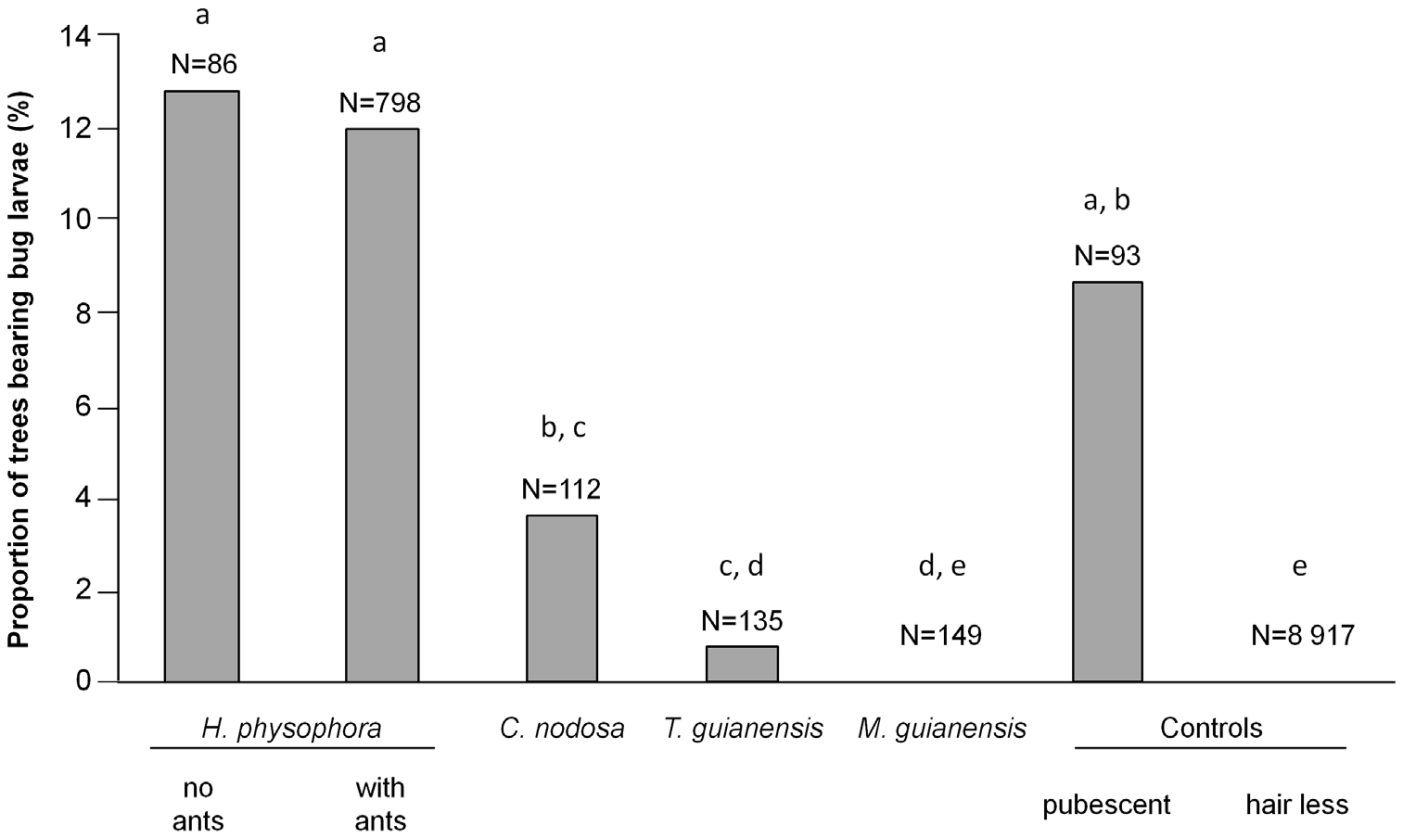
Distribution of *Z. annulosus* on trees. Proportion of trees bearing *Zelus annulosus* in each category. N = number of plants observed; different letters indicate significant differences at *P*<0.01 (Fisher’s exact-tests and the sequential Bonferroni procedure). The four *Cordia nodosa* and the *Tococa guianensis* sheltering *Z. annulosus* were occupied by *Allomerus octoarticulatus* and *Crematogaster laevis*, respectively, while the other treelets sheltered *Azteca* spp. colonies.

The 405 *Z. annulosus* recorded in total were found on 120 pubescent plants, including some myrmecophytes (Fig. 1). This corresponded to a mean (±se) of 3.37±0.30 nymphs per plant. We did not record *Z. annulosus* individuals on *M. guianensis* or on hairless control plants. The nymphs of all instars were noted on *H. physophora* whether or not they were sheltering an *A. decemarticulatus* colony as well as on pubescent plants (non-significant differences between the three groups of plants; Fig. 1). Conversely, we noted significantly more *Z. annulosus* individuals on *H. physophora* with ants than on *C. nodosa* or *T. guianensis* that all sheltered an ant colony (Fig. 1).

**Figure 2:**
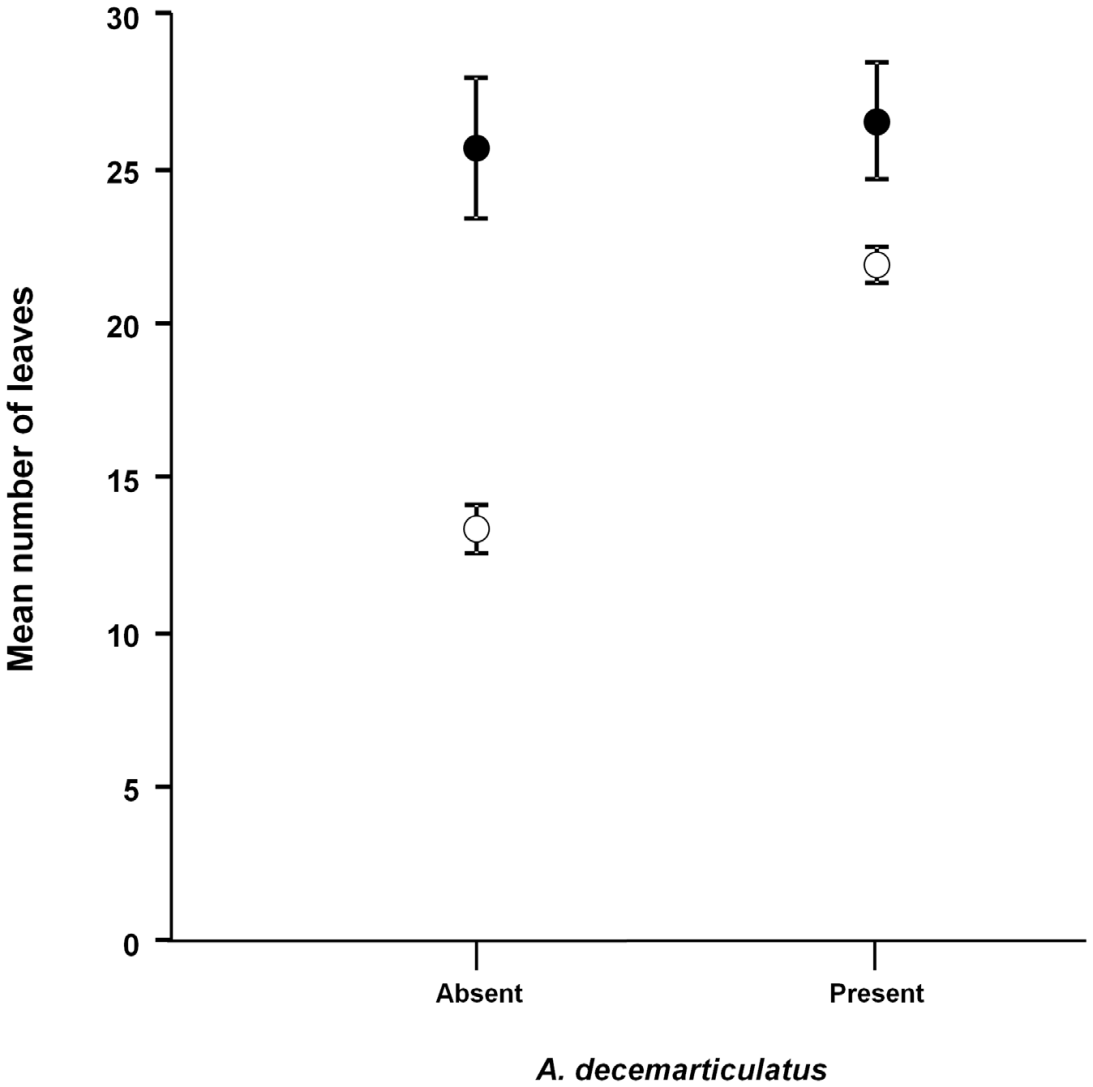
Factors influencing the presence of *Z. annulosus* on *Hirtella physophora* trees. Illustration of the presence of *Z. annulosus* as a function of the presence of *Allomerus decemarticulatus* and the mean number of leaves (±se) of *H. physophora*. Presence of *Z. annulosus* (filled circle), absence (empty circle). GLM: Factors ants: *ns*; size: *ns*; leaves: *P*<0.01; ants-size interaction: *ns*; leaves-size interaction: *ns*; ants-leaves interaction: *P*<0.01.

All stages of the *Z. annulosus* life cycle (i.e., egg clusters, nymphs and adults) were recorded on *H. physophora* and non-myrmecophytic pubescent plants. The GLM analysis confirmed that *Z. annulosus* were found on *Hirtella physophora* individuals whether or not they were sheltering ants (GLM, factor “ants”: *ns*; Fig. 2); however, these trees have significantly more leaves than those without *Z. annulosus* nymphs (GLM, factor “leaves”: *P* = 0.00145). The interaction between the presence of ants and the number of leaves was also significant (GLM, factors “ants presence:leaves”: *P* = 0.00269).

A series of seven surveys was conducted to examine the frequency of occupation of certain *H. physophora* individuals by *Z. annulosus*. We noted that 33 treelets never sheltered *Z. annulosus* nymphs or egg clusters, 18 others sheltered *Z. annulosus* two to four times, and the 14 remaining treelets sheltered nymphs or egg clusters during all of the surveys or all but one.

### Field observations on plant x *Zelus annulosus* x *Allomerus decemarticulatus* relationships

During the 27 non-consecutive hours of observation (15 min for each of the 107 *H. physophora* bearing at least one *Z. annulosus* individual; 363 *Z. annulosus* observed), *Z. annulosus* nymphs never extended their rostrum towards extrafloral nectaries nor did they suck the sap from the leaves or young parts of the stem. Most of the time, the first instar nymphs rested beneath the leaves, while those of the later instars rested on the surface of the leaves or adopted an ambushing posture with their raised anterior legs spread open. They hid under the leaves when disturbed, before it rained, or in order to moult.

Although we observed *Z. annulosus* nymphs capturing or having captured 48 insect prey (i.e., winged termites, cockroaches, orthopterans, dipterans and coleopterans), we never saw them ambushing *A. decemarticulatus* workers to capture them or trying to rob their prey. During occasional encounters with patrolling workers, *Z. annulosus* individuals only raised their legs, probably to avoid contact with the ants, while the latter generally ignored their presence or did not detect them and never recruited nestmates to evict them. Nevertheless, in three cases the ants came into contact with a leg extremity and bit it, and were immediately glued to its sticky surface. After a brief attempt to escape, the bitten *Z. annulosus* bit the ant in turn and fed on it.

When *Z. annulosus* nymphs moved from one leaf to another they walked on the upper part of the stems, avoiding putting their legs on the trap where *A. decemarticulatus* workers ambushed in a group. Nevertheless, there were exceptions as five first and second instar nymphs were captured, meaning that exceptionally small *Z. annulosus* nymphs can be caught despite the care they take to avoid the traps.

### Experiments on the relationship between *Zelus annulosus* nymphs and *Allomerus decemarticulatus* workers

When termite prey were experimentally placed onto the centre of the leaves, *Z. annulosus* nymphs always detected them first, captured them and fed on them, while *A. decemarticulatus* workers never tried to rob these prey. If a patrolling worker arrived by chance, the *Z. annulosus* only lifted the prey with its rostrum, avoiding any contact between the prey and the ant (N = 17 cases out of 50).

When prey were dropped near the domatia, *A. decemarticulatus* and *Z. annulosus* caught them first 20 and 30 times, respectively (Fisher's exact-test: *P* = 0.07). When the ants caught the prey first, *Z. annulosus* nymphs approached the domatia in five cases out of 20, extending their rosters toward the prey, but finally gave up and moved further away. The *A. decemarticulatus* workers never reacted when the *Z. annulosus* nymphs caught the prey first, allowing the nymphs to move slowly toward the centre of the leaves to eat the prey.

When prey were placed on the stems, *A. decemarticulatus* workers were always the first to find and spread-eagle them; *Z. annulosus* nymphs never tried to feed on these prey.

### Effectiveness of the predatory behaviour by *Z. annulosus* nymphs

Both small and large nymphs captured termites easily as only one termite prey was abandoned in each case. Concerning grasshopper capture, six individuals escaped by jumping away, while in all of the other cases the grasshoppers were captured although they struggled (86.67% of the successful captures).

## Discussion

### Selection of the host plant

Host plant selection in *Z. annulosus* is not related to the food provided by the host plant or to insect prey specifically associated with that host plant, nor do the nymphs of this species gather the sticky substance that coats their legs from certain plants, but instead they secrete it [4], [16], [20], [23].

Nevertheless, selection undoubtedly occurs as *Z. annulosus* individuals and egg clusters were recorded on “pubescent plants”, including *H. physophora* treelets whether or not they were sheltering an *A. decemarticulatus* colony. By selecting pubescent plants for oviposition, *Z. annulosus* females permit their offspring to be exposed to only small ant species because larger ants are reluctant to patrol these plants [30]. As *Z. annulosus* nymphs frequently walk on the trichomes rather than on the leaf surface, their legs are rarely in direct contact with the small ants that patrol just below them, between the trichomes. The difference in size also permits the nymphs to avoid being attacked by small ants by raising the leg or legs likely to be discovered by patrolling workers, or by moving as these ants are very slow. On the contrary, hairless plants are often patrolled by larger ants able to capture nymphs or to destroy egg clusters [26], [30].

Nevertheless, although pubescent, other myrmecophytes are mostly or even completely avoided. The exceptions are when *C. nodosa* shelters *A. octoarticulatus* and *T. guianensis* shelters *Crematogaster laevis* (Fig. 1). All other *C. nodosa* and *T. guianensis* individuals shelter *Azteca* spp. colonies whose workers are much bigger and faster in their movements than are those of *Allomerus* spp. or *C. laevis*. They can attack *Z. annulosus* females trying to oviposit on their host plant, or prey on an egg cluster or nymphs if oviposition was successful. The absence of *Z. annulosus* on *M. guianensis* is likely due to predation pressure by their associated plant-ants, *Pheidole minutula*, whose soldiers are able to destroy the egg clusters or to capture first instar nymphs [31].

As adults leave their host plants (probably to mate), the high frequency of occupation observed on some *H. physophora* individuals could be the result of the selection of particular micro-climatic factors or intrinsic plant factors favourable to the development of offspring.

### *Zelus annulosus* relationships with host plants and *Allomerus*

The plants probably gain protection from potential defoliators as *Z. annulosus* nymphs are good predators, including when confronted with grasshoppers likely to escape by jumping away. When sheltering on *H. physophora* that are already protected by their mutualist ants, *Z. annulosus* acts as a second line of defence. Indeed, whereas *A. decemarticulatus* workers recruit nestmates mainly after contact with alien insects that have damaged leaf blades [32], *Z. annulosus* nymphs detect prey visually (even from one leaf to another) and immediately capture them. As *Z. annulosus* nymphs never feed at the expense of their host plants by sucking sap or feeding on nectar, the presence of trichomes seems to be the unique advantage that they gain from the plants. The trichomes of pubescent plants are a constitutive protection from insect herbivores by acting as a physical barrier denying larger herbivorous insects access to leaves [33], [34]. However, this protection can turn against the plant by forming an enemy-free space hampering the access of parasitoids to the smaller herbivorous insects able to filter between them [35]–[37]. On *H. physophora*, trichomes offer an enemy free space to *Z. annulosus* by providing protection from larger ants as well as from the smaller *A. decemarticulatus,* but is not detrimental to the plant as in a parasitoid-herbivore enemy-free space. Therefore, both the host plant and *Z. annulosus* benefit slightly from the association: the trichomes on pubescent plants protect *Z. annulosus* nymphs from large, predatory ants whereas the host plants are somewhat protected from defoliating insects. The GLM analysis shows that the large number of leaves is related to ant presence. Actually, plant-ants are a constitutive defence for ant-plants and preserve leaves from herbivores [38]. Moreover, some plant-ants are known to castrate their host-tree, which in return produces more leaves due to a phenomenon of resource re-allocation from the reproductive parts to the vegetative parts [39]. The fact that *H. physophora* bearing *Z. annulosus* had more leaves than trees without *Z. annulosus* is unlikely due to the protection provided by the bugs as the trees do not continually shelter them. The presence of bug nymphs on trees with a large number of leaves is probably the consequence of the oviposition choice made by females.

It is likely that *Z. annulosus* females were not repelled by the presence of an *A. decemarticulatus* colony during oviposition because we noted similar percentages of nymphs on *H. physophora* treelets whether or not they sheltered a colony of this ant species (the same is probably true for *A. octoarticulatus* on *Cordia nodosa*). Although accidental captures of both the workers and small bug nymphs were noted, these species mostly share the *H. physophora* treelets rather peacefully; for example, the areas surrounding the domatia seem to be where *A. decemarticulatus* and *Z. annulosus* compete for prey, but we never noted them fighting over prey. The small size and the slow movements of the *A. decemarticulatus* workers permit *Z. annulosus* nymphs to avoid them, which is especially easy as they hunt mainly on large, mature leaves that are patrolled only from time to time by two to four workers [32]. Like for social wasps [12], the *A. decemarticulatus* colonies can provide *Z. annulosus* nymphs protection from army ants (or other predators coming from the ground), while receiving nothing in return. Therefore, *A. decemarticulatus* colonies and *Z. annulosus* individuals share the same habitat even though they are somewhat in competition. The number of reciprocal captures was so low that we cannot speak of intraguild predation or the killing and eating of species that use similar resources and so are potential competitors [27]. This relationship differs therefore from cases of Holoptilinae specialized in ant predation [22] or from kleptoparasitism where social wasps rob prey from plant-ants [40].

To summarize, we found *Z. annulosus* only on pubescent plants, including the myrmecophyte *H. physophora* whether or not the plant was sheltering a mutualistic *A. decemarticulatus* colony. The plant is slightly protected from defoliators, while *Z. annulosus* is aided throughout its entire development. The relationship between *Z. annulosus* and *A. decemarticulatus* corresponds to the simple coexistence of two competitors sharing hunting areas, with *Z. annulosus* individuals benefiting from enemy-free space thanks to the presence of plant trichomes protecting them from *A. decemarticulatus* attacks.
